# Τhe Impact of Pre-Transplant Kidney Biopsy on the Evaluation of Prospective Living Kidney Donors

**DOI:** 10.3390/jcm12072685

**Published:** 2023-04-04

**Authors:** Smaragdi Marinaki, Kalliopi Vallianou, Maria Darema, Evangelos Mantios, Eleni Kapsia, Christina Melexopoulou, Vassilis Filiopoulos, George Liapis, Ioannis N. Boletis

**Affiliations:** 1 Clinic of Nephrology and Renal Transplantation, Laiko General Hospital, Medical School of Athens, National and Kapodistrian University, 11527 Athens, Greece; 2 1st Department of Pathology, Medical School of Athens, National and Kapodistrian University, 11527 Athens, Greece

**Keywords:** renal transplantation, living donation, renal biopsy, marginal donors

## Abstract

Living kidney donation contributes to increasing the donor pool. Since safety and excellent outcomes of living kidney donors (LKD) are essential, renal biopsy must be part of the pre-transplant evaluation in donors with isolated urine abnormalities or other risk factors. We retrospectively collected data on potential living donors evaluated in the pre-transplant outpatient clinic of Laiko General Hospital of Athens between 2007 and 2022, who underwent a pre-transplant biopsy. Biopsy indications included microscopic hematuria, borderline proteinuria and comorbidities suggestive of chronicity. Those with glomerular diseases or chronic lesions were excluded from donation. We identified 59 potential living donors who underwent renal biopsy. Of these, 10 (16.9%) were male. Median age was 58 (IQR 51–63) years, while 23 (39%) were older than 60 years. 49 out of 59 (83%) had glomerular hematuria, 10 (16.7%) had proteinuria (150–300 mg/d). Out of the 59 donors, 21 (35.6%) were hypertensive, three (5.1%) had impaired glucose tolerance and seven (11.9%) had a BMI > 30 kg/m^2^. A total of 32 (54.2%) potential donors were accepted for donation. Eight (13.6%) had IgA nephropathy, 10 (16.9%) TBMD and nine (15.3%) had increased chronicity including secondary FSGS. When compared with a control group of donors who did not need a pre-transplant biopsy, those 32 who donated were more frequently hypertensive (*p* = 0.003), but had similar eGFR [61.3 (±10.4) vs. 61.9 (±13.8), *p* = 0.866] after a follow-up of 79 (36–114) months. Renal biopsy is a useful tool in the evaluation of prospective LKD. Thorough assessment of donors with isolated urine abnormalities and marginal donors is critical to ensure good post-donation outcomes.

## 1. Introduction

Kidney transplantation is the treatment of choice for end-stage kidney disease (ESKD); however, a lack of organs is the major impediment towards this treatment direction [1], and therefore living kidney donation increases the donor pool. Out of the 23.853 transplantations performed in the USA in 2020, 22.2% were from a living donor [2], while the European Renal Association (ERA) registry reports 18.136 transplantations in 2020, 28% of which were from a living donor [3]. In Greece, the contribution of living donation is even more significant, as 1185 patients are currently on the waiting list; the mean waiting time is 8.8 years and only 9 transplantations per million population were performed in 2020 [3]. As donor safety is of utmost importance, an extensive pre-donation evaluation is required. Donor factors such as hematuria of glomerular origin, borderline proteinuria, marginal kidney function and the presence of comorbidities predisposing to chronic kidney disease necessitate further evaluation for the donor’s safety and favorable long-term outcome [4]. Microscopic hematuria and mildly elevated proteinuria are common, affecting 4–5% [5] and 2.4% [6] of the general population, respectively. After excluding extra-renal causes of hematuria, a kidney biopsy is required to diagnose an underlying kidney disease, especially in the presence of comorbidities such as hypertension, diabetes and obesity [4].

Herein, we aimed to analyze the characteristics of prospective living kidney donors who underwent a pre-transplant kidney biopsy, demonstrate how the biopsy facilitates the decision towards living kidney donation, and address whether this is associated with positive donor outcomes.

## 2. Materials and Methods

Data from potential living kidney donors evaluated at the pre-transplant outpatient clinic of the Laiko General Hospital of Athens between 2007 and 2022 were retrospectively collected. Individuals in need of a pre-transplant kidney biopsy were identified. Data on post-donation follow-up of the donors, who attend the living donor outpatient clinic, were collected as well. The study was approved by the Institutional Review Board of the Laiko General Hospital (protocol number: 213, 20/1/2023) and was conducted in accordance with the Declaration of Helsinki.

Microscopic hematuria was defined by the presence of more than three red blood cells per high-power field in three unrelated evaluations in the absence of urinary tract infection. Glomerular origin of hematuria was assessed by urine microscopy. Non-glomerular hematuria was further investigated for urinary tract infection, lithiasis, tumor or anatomical variations. Proteinuria was assessed by two unrelated 24 h urine collections in the absence of infection, fever or exercise. Borderline proteinuria was defined as daily urine protein excretion of 150–300 mg. Glomerular filtration rate (GFR) was estimated by the CKD-EPI (Chronic Kidney Disease-Epidemiology Collaboration) formula and measured by radionuclides. Potential donors with a GFR ≥ 90 mL/min/1.73 m^2^ were accepted for donation and those with GFR ≤ 60 mL/min/1.73 m^2^ were rejected, as recommended by the KDIGO guidelines [4]. When GFR was between 60 and 89 mL/min/1.73 m^2^, the advisory tables of the British Transplantation Society were used to determine the acceptable preoperative GFR by age and gender [7]. A kidney biopsy was performed in potential donors exhibiting persistent microscopic hematuria of glomerular origin and/or borderline proteinuria, and in potential donors with comorbidities such as long history of hypertension, on antihypertensive treatment with more than two drugs, impaired glucose tolerance or abnormal HbA1c, BMI > 30 kg/m^2^ or any combination of the above. Kidney biopsy was assessed by light microscopy, immunofluorescence and electron microscopy. Potential donors diagnosed with IgA nephropathy, secondary FSGS, global glomerulosclerosis, severe interstitial fibrosis and tubular atrophy, severe arteriolar hyalinosis and arteriosclerosis or thin basement membrane nephropathy (glomerular basement membrane thickness of <200 nm) (TBMN) were excluded from donation. Individuals whose glomerular basement membrane thickness was between 200 and 250 nm underwent a genetic analysis testing for mutations in COL4A3, COL4A4 and COL4A5 genes.

Prospective donors were evaluated by a team consisting of transplant nephrologists and a transplant surgeon. Extended-criteria potential donors were accepted after thorough evaluation. Donors were followed in the living donor outpatient clinic three months post-donation and then every 6–12 months.

Donors who underwent a pre-transplant kidney biopsy were compared with a control group of gender- and age-matched donors who did not need a pre-transplant biopsy, with a ratio of 1:4. Statistical analysis was performed using STATA 14 (StataCorp LLC, College Station, TX, USA). Categorical variables are expressed as frequencies and percentages. Numerical variables following a normal distribution are expressed as means and standard deviation (SD), whereas skewed numerical variables are expressed as median and interquartile range (IQR). Differences were calculated using a chi-square test for categorical variables, Student’s *t*-test for normally distributed numerical variables and Mann–Whitney U test for skewed numerical variables. Significance was defined as *p* < 0.05.

## 3. Results

**Figure 1:**
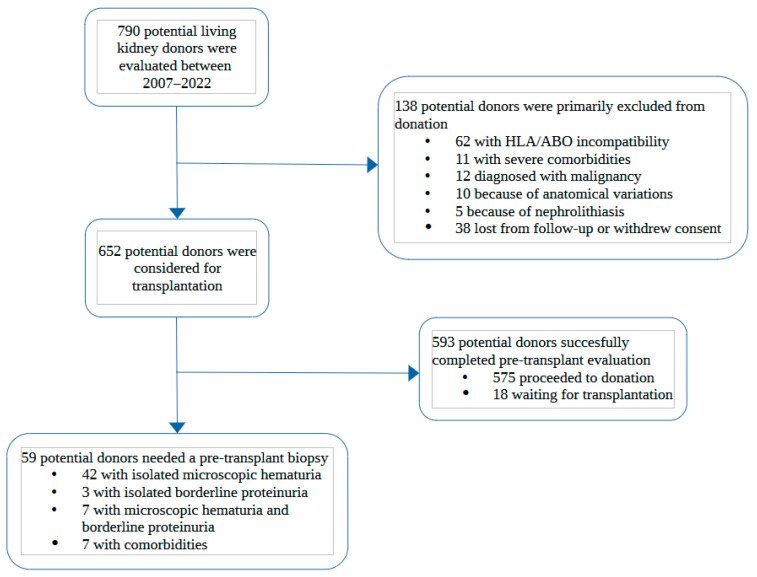
Flowchart describing the kidney donor selection process from initial evaluation in the outpatient clinic to final decision on pre-transplant biopsy. HLA: Human Leucocyte Antigens; comorbidities: long history of hypertension, on ≥3 antihypertensive drugs, impaired glucose tolerance or HbA1c, BMI > 30 kg/m^2^.

Among 790 potential living kidney donors, we identified 59 potential donors who underwent a pre-transplant kidney biopsy. The donor selection process is described in Figure 1.

### 3.1. Characteristics of Potential Living Kidney Donors and Pre-Transplant Biopsy Indications

The indications to perform a kidney biopsy were isolated microscopic hematuria of glomerular origin in 42 out of 59 (71.1%) potential donors, isolated borderline proteinuria in 3 out of 59 (5.1%), glomerular hematuria and borderline proteinuria in 7 out of 59 (11.9%) and history of the aforementioned comorbidities in 7 out of 59 (11.9%). The majority (83.1%) were women, and their median age was 58 (IQR 51–63) years, with 23 (39%) being older than 60 years old. All potential donors were Caucasian. Out of 59 potential donors, 21 (35.6%) were diagnosed with hypertension, 7 (11.9%) were obese with a BMI > 30 kg/m^2^ and 3 (5.1%) had impaired glucose tolerance. The mean creatinine level was 0.76 (±0.14) mg/dL and eGFR was 90.8 (±13.6) mL/min/1.73 m^2^, similar to mGFR [90.3 (±17.5) ml/min/1.73 m^2^]. Demographic characteristics, comorbidities and laboratory findings of potential donors classified according to biopsy indication are demonstrated in Table 1.

Individuals with isolated microscopic hematuria were more likely to have normal biopsy findings (52.4%) and TBMN (23.8%). Individuals with borderline proteinuria alone or with microscopic hematuria were older, more likely to have comorbidities and abnormal biopsy findings (chronic lesions in 40% and IgA nephropathy in 20%). On the other hand, the majority (85.7%) of potential donors with comorbidities, but no urine abnormalities, had minimal biopsy findings.

### 3.2. Biopsy Results and Exclusion from Donation

Potential donors are also categorized according to their kidney biopsy findings: those without obvious histologic alterations allowing them to donate, those diagnosed with IgA nephropathy, TBMN, and those with chronic lesions including secondary FSGS. The data on demographics, kidney function, urine abnormalities and aforementioned biopsy findings are provided in Table 2.

Out of 59 potential donors, 32 (54.2%) did not demonstrate substantial histologic findings and were accepted for donation. Out of these 32, 12 (37.5%) had hypertension, 4 (12.5%) were obese and 2 (6.3%) were glucose intolerant. A total of 24 out of 49 donors with hematuria (49%) and 4 out of 10 donors (40%) with borderline proteinuria had normal biopsy findings. Six (18.8%) had glomerular basement membrane thickness between 200 and 250 nm and proceeded to undergo genetic analysis testing, which was negative for mutations in COL4A3, COL4A4 and COL4A5 genes. The remaining 27 out of the 59 (45.7%) potential donors were excluded from donation. We identified eight individuals with IgA nephropathy and ten with TBMN, all exhibiting glomerular hematuria but excellent kidney function, and who were referred to the glomerulonephritis outpatient clinic in our department for further follow-up. We also identified nine individuals exhibiting increased chronic lesions (severe interstitial fibrosis and tubular atrophy, severe arteriolar hyalinosis and arteriosclerosis) and/or secondary FSGS lesions.

### 3.3. Post-Donation Follow-Up of Living Kidney Donors Who Underwent a Pre-Transplant Biopsy

After a median follow-up of 79 (34–116) months, the 32 living kidney donors who donated after a pre-transplant biopsy had satisfactory post-donation renal function [creatinine 1.08 (±0.14)-eGFR 61.3 (±13.8) ml/min/1.73 m^2^] and a median proteinuria of 77 (62–164) mg/d. In Table 3, they are compared with a control group of age- and gender-matched donors who did not need a pre-transplant biopsy.

Hypertension was significantly more frequent in donors who needed a pre-transplant biopsy (37.5% vs. 18.7%, *p* = 0.003). They also had a higher BMI and lower eGFR and were more likely to smoke; however, these findings were not of statistical significance. Nonetheless, kidney function and daily urine protein excretion were comparable in the two groups after a similar follow-up period.

## 4. Discussion

Herein, we describe 59 potential living kidney donors who underwent a pre-transplant kidney biopsy. Microscopic glomerular hematuria was the most common indication for performing a kidney biopsy (71.1%), followed by borderline proteinuria and/or hematuria (17%) and the presence of comorbidities (11.9%). About 40% of them were older than 60 years old and one third of them were hypertensive. Despite this, 32 out of 59 (54.2%) had minimal kidney biopsy findings and proceeded to donation. The different diagnoses associated with each biopsy indication show that diagnosis may be presumed by medical history, but can only be confirmed by tissue biopsy. Therefore, it is important to perform a kidney biopsy for every potential donor with urine abnormalities and to not accept or reject them solely based on medical history or laboratory findings.

We found that almost half of the potential donors were rejected based on biopsy findings. In the literature, four studies describe rejection rates of 38–60% [8,9,10,11]. However, in two studies, the biopsy indication was isolated microscopic hematuria [8,10]. In another two studies, the indications were hematuria, borderline proteinuria and prediabetes as well [9,11]. Hassan et al. included 45 potential donors with isolated microscopic hematuria and rejected 38% [8]. Contrary to our population, they were 32.6 ± 8 years old, normotensive and had no proteinuria. The remaining studies included fewer potential donors: 6, 10 and 15, respectively [9,10,11]. We performed a pre-transplant kidney biopsy in every potential donor with isolated microscopic hematuria, even in the absence of proteinuria, hypertension or marginal kidney function, and rejected those with evidence of glomerular disease. A similar approach was the one described by Hassan et al., where among 45 potential donors with isolated microscopic hematuria who underwent a kidney biopsy between 2010 and 2016, 38% had TBMN or IgA nephropathy, while the remaining 62% proceeded to donation [8]. Choi et al. and Koushik et al. reported the same results in smaller samples [10,11]. Conversely, Kido et al. reviewed the records of 242 donors between 2001 and 2007. Pre-donation glomerular hematuria was found in 15 of them and was strongly associated with the development of proteinuria and a faster decline in kidney function. The authors suggested that donors with persistent glomerular hematuria should be excluded from kidney donation [12]. Van der Weijden et al. recently suggested that potential donors with mild isolated hematuria without other risk factors could donate without a pre-transplant biopsy. The authors found that among 701 donors, 88 had pre-donation hematuria, which was not associated with a post-donation decline in GFR, proteinuria or hypertension after a median follow-up of 5 years. However, they proposed a pre-transplant biopsy in those with hypertension, marginal proteinuria or positive family history for kidney disease [13]. Currently, we prefer to perform a pre-transplant biopsy to evaluate every potential donor with glomerular hematuria, considering the minimal complications of the procedure [14].

We found that 8 out of the 59 potential donors had IgA nephropathy and 10 had TBMN. These two diagnoses are the most commonly encountered in people with isolated glomerular hematuria [8,10,15]. Interestingly, IgA nephropathy and TBMN can coexist, as described in a case by Vadivel et al. [16]. All of our potential donors finally diagnosed with IgA nephropathy had microscopic hematuria, whereas two had microscopic hematuria and marginal proteinuria. We excluded all of them from donation, as is generally accepted, considering that IgA nephropathy’s clinical course is variable and unpredictable [17].

We excluded all 10 potential donors diagnosed with TBMN as well. Despite their excellent kidney function and the absence of proteinuria and hypertension, we do not consider donation safe in this population in the long-term. It is generally accepted that female carriers of X-linked Alport syndrome do not donate because of the well-established risk of chronic kidney disease, proteinuria and hypertension usually in older age. Indeed, Gross et al. described that kidney function significantly declined in four out of six donors after an average 6.7 years post-donation and underlined the increased risk of kidney failure [18]. On the other hand, the rejection of potential donors with TBMN has not been standard practice but a subject of debate in the literature. Choi et al. mention excellent mid-term outcomes in 11 donors with TBMN for a mean follow-up of 57.4 months post-donation [19]. However, heterozygous mutations in COL4A3 or COL4A4 lead to various phenotypes in the spectrum of Alport syndrome and TBMN [20]. In a literature review by Savige et al., hypertension is present in 17% and proteinuria > 500 mg/d in 16% of individuals with TBMN [21]. Impaired kidney function is present in up to 7–15%, usually in older individuals [21,22]. Although KDIGO guidelines of 2017 are inconclusive on whether to accept potential donors with TBMN [4], the latest 2021 Guidelines for Genetic Testing and Management of Alport Syndrome of the American Society of Nephrology advise against donation and underline the high risk of kidney impairment due to COL4A3/COL4A4 mutation and further deterioration of kidney function post-donation [23].

In our center, 112 transplantations were performed in 2022, 65 (58%) of which were from a living donor. Considering graft shortage, we are making the most of grafts from marginal donors: older, hypertensive, obese, and with prediabetes. In such cases, we perform a pre-donation kidney biopsy to evaluate tissue damage. Nine out of the fifty-nine potential donors that underwent a pre-transplant biopsy in our study were excluded from donation because of the presence of chronic lesions, such as FSGS lesions, severe interstitial damage, arteriosclerosis and arteriolar hyalinosis. Indeed, Kakuta et al. reported that, among 1351 donors, those with arteriosclerosis had significantly lower GFR 10 years post-donation and advised against donation in the presence of more severe arteriosclerosis [24]. Moreover, Merzkani et al. found that, among 950 donors, those with controlled hypertension had larger glomeruli and arteriolar hyalinosis in implantation biopsies, but did not have significantly lower GFR 10 years post-donation. However, high office blood pressure and non-dipping status were associated with higher risk of proteinuria [25]. Fahmy et al. followed 310 donors who underwent an implantation protocol biopsy for a median of 6.2 years. They found that among histological abnormalities, only IFTA was associated with significantly decreased GFR post-donation. Lower pre-donation GFR and older age were associated with lower post-donation GFR as well [26]. No pre-donation histological feature or degree of tissue damage can consistently predict post-donation outcomes in potential donors with risk factors, particularly hypertension. Therefore, we individualize every case. Recipient factors, such as age, time on dialysis and possible dialysis complications are considered, as well as the absence of an alternative donor, since mean time on the waiting list for a deceased donor is almost 9 years. High-risk prospective donors are thoroughly informed about possible long-term renal complications, and the strong desire to donate is always taken into account.

Our study has several limitations, including the retrospective design (facilitating information and selection bias), and the small number of potential donors which complicates the interpretation of our results. Additionally, because of the population’s heterogeneity regarding biopsy indications, it is difficult to associate clinical and histological findings. Moreover, a longer follow-up of the donors would be useful. Finally, it would be interesting to study the outcomes of those rejected from donation, since they are followed in the glomerulonephritis and CKD outpatient clinic.

## 5. Conclusions

In conclusion, we have demonstrated that pre-transplant kidney biopsy is a useful tool in the evaluation of prospective living kidney donors. We have utilized 32 donors with urinary abnormalities and/or risk factors that we would have rejected without a biopsy. The need to utilize every possible living donor is imperative because of the imbalance between organ demand and supply, and the lack of deceased donors. On the other hand, we have rejected 27 potential donors with glomerular disease and/or chronicity, who probably would have developed advanced CKD if they had donated. Short- and mid-term outcomes of carefully selected donors with isolated urinary abnormalities and marginal donors are comparable to donors with a normal pre-transplant evaluation who did not need a pre-transplant biopsy. The long-term safety and efficacy remain to be analyzed in future research.

## Figures and Tables

**Table 1:** Demographic and clinical characteristics of potential living kidney donors who underwent a pre-transplant kidney biopsy, classified by biopsy indication [N (%) or Mean (±SD) or Median (IQR)].

	Total Potential Donors N = 59	Individuals with Isolated Microscopic Hematuria N = 42	Individuals with Borderline Proteinuria ± Hematuria Ν = 10	Individuals with Comorbidities N = 7
Gender (male)	10 (16.9)	4 (9.5)	5 (50)	1 (14.3)
Age (years)	58 (51–63)	56.5 (50.3–61)	62.5 (56.7–67)	64 (52–67)
Older than 60 years old	23 (39)	12 (28.6)	6 (60)	5 (71.4)
Hypertension	21 (35.6)	8 (19)	7 (70)	6 (85.7)
On antihypertensive medication 1 drug 2 drugs 3 drugs	21 (35.6) 16 (27.1) 4 (6.8) 1 (1.7)	8 (19) 7 (16.7) 1 (2.4)	7 (70) 4 (40) 2 (20) 1 (10)	6 (85.7) 5 (71.4) 1 (14.3)
BMI (kg/m^2^)	27 (24.6–29.1)	26 (24–27.5)	27.3 (25.9–29.7)	29.4 (28.3–31.5)
BMI > 30 (kg/m^2^)	7 (11.9)	2 (4.8)	2 (20)	3 (42.9)
Smoking	17 (28.8)	9 (21.4)	4 (40)	4 (57.1)
Abnormal OGTT	3 (5.1)	0 (0)	2 (20)	1 (14.3)
Creatinine (mg/mL)	0.76 (±0.14)	0.74 (±0.13)	0.87 (±0.15)	0.81 (±0.16)
eGFR (mL/min/1.73 m^2^)	90.8 (±13.6)	93 (±11.9)	82.2 (±13.1)	88.7 (±14.9)
eGFR < 80 mL/min/1.73 m^2^	11 (18.6)	6 (14.3)	4 (40)	1 (14.3)
mGFR (mL/min/1.73 m^2^)	90.3 (±17.5)	92.2 (±18.8)	81.4 (±14.9)	86.7 (±15.2)
mGFR < 80 mL/min/1.73 m^2^	11 (18.6)	6 (14.3)	4 (40)	1 (14.3)
Diagnosis:				
Minimal findings	32 (54.2)	22 (52.4)	4 (40)	6 (85.7)
IgA nephropathy	8 (13.6)	6 (14.3)	2 (20)	0 (0)
TBMN	10 (16.9)	10 (23.8)	0 (0)	0 (0)
Chronic lesions	9 (15.3)	4 (9.5)	4 (40)	1 (14.3)

BMI: body mass index, OGTT: oral glucose tolerance test, eGFR: estimated glomerular filtration rate, mGFR: measured glomerular filtration rate, TBMN: thin basement membrane nephropathy.

**Table 2:** Demographic and clinical characteristics of potential living kidney donors who underwent a kidney biopsy classified by diagnosis [N (%) or Mean (±SD) or Median (IQR)].

	Total Potential Donors N = 59	Individuals Accepted for Donation N = 32	Individuals Diagnosed with IgA Nephropathy Ν = 8	Individuals Diagnosed with TBMN N = 10	Individuals Diagnosed with Secondary FSGS-Increased Chronicity N = 9
Gender (male)	10 (16.9)	4 (12.5)	2 (25)	0 (0)	4 (44.4)
Age (years)	58 (51–63)	60.5 (50–65.3)	53.5 (51–55)	59.5 (47.3–60.8)	58 (57–63)
Older than 60 years old	23 (39)	15 (46.7)	1 (12.5)	4 (40)	3 (33.3)
Hypertension	21 (35.6)	12 (37.5)	3 (37.5)	1 (10)	3 (37.5)
On antihypertensive medication 1 drug 2 drugs 3 drugs	21 (35.6) 16 (27.1) 4 (6.8) 1 (1.7)	12 (37.5) 9 (28.1) 3 (9.4)	3 (37.5) 3 (37.5)	1 (10) 1 (10)	5 (55.5) 3 (33.3) 1 (11.1) 1 (11.1)
BMI (kg/m^2^)	27 (24.6–29.1)	27 (24.8–29)	26 (25.5–27.1)	24.4 (23.8–25.3)	29.5 (27.3–30.9)
BMI > 30 (kg/m^2^)	7 (11.9)	4 (12.5)	0 (0)	1 (10)	2 (22.2)
Smoking	17 (28.8)	9 (28.1)	2 (25)	2 (20)	4 (44.4)
Abnormal OGTT	3 (5.1)	2 (6.3)	0 (0)	0 (0)	1 (11.1)
Creatinine (mg/dL)	0.76 (±0.14)	0.73 (±0.14)	0.79 (±0.08)	0.7 (±0.08)	0.92 (±0.13)
eGFR (mL/min/1.73 m^2^)	90.8 (±13.6)	91.3 (±13.8)	94.2 (±9.6)	97.3 (±11.9)	80.2 (±11.4)
eGFR <80 mL/min/1.73 m^2^	11 (18.6)	5 (15.6)	1 (12.5)	1 (10)	4 (44.4)
mGFR (mL/min/1.73 m^2^)	90.3 (±17.5)	91.3 (±18.2)	91.5 (±15.3)	93 (±11.2)	75.3 (±13.2)
mGFR < 80 mL/min/1.73 m^2^	11 (18.6)	5 (15.6)	1 (12.5)	1 (10)	4 (44.4)
Glomerular hematuria	42 (71.1)	22 (68.8)	6 (60)	10 (100)	4 (44.4)
Borderline proteinuria (150–300 mg/d)	3 (5.1)	2 (6.3)	0 (0)	0 (0)	1 (11.1)
Hematuria and borderline proteinuria	7 (11.9)	2 (6.3)	2 (25)	0 (0)	3 (33.3)

BMI: body mass index, OGTT: oral glucose tolerance test, eGFR: estimated glomerular filtration rate, mGFR: measured glomerular filtration rate, TBMN: thin basement membrane nephropathy, FSGS: focal segmental glomerulosclerosis.

**Table 3:** Demographics, comorbidities and outcomes of living kidney donors who donated with and without a pre-transplant biopsy [N (%) or Mean (±SD) or Median (IQR)].

	Donors Accepted without a Pre-Transplant Biopsy N = 128	Donors Who Underwent a Pre-Transplant Biopsy N = 32	*p*-Value
Gender (male)	16 (12.5)	4 (12.5)	0.863
Age (years)	61 (50.5–66)	60.5 (50–65.3)	0.628
Hypertension	24 (18.7)	12 (37.5)	0.003
BMI (kg/m^2^)	26.1 (24–28.1)	27 (24.8–29)	0.139
Smoking	24 (18.8)	9 (28.1)	0.202
Abnormal OGTT	7 (5.5)	2 (6.3)	0.511
Creatinine (mg/dL)	0.7 (±0.16)	0.73 (±0.13)	0.537
eGFR (mL/min/1.73 m^2^)	96.2 (±17.1)	91.3 (±13.8)	0.103
Glomerular hematuria	0 (0)	24 (75)	<0.001
Borderline proteinuria (150–300 mg/d)	0 (0)	4 (12.5)	<0.001
Follow up (months)	80 (34–116)	79 (36–114)	0.725
Creatinine at follow-up (mg/dL)	1.08 (±0.18)	1.08 (±0.14)	0.736
eGFR at follow-up (mL/min/1.72 m^2^)	61.9 (±13.8)	61.3 (±10.4)	0.866
Proteinuria at follow-up (mg/d)	88.5 (75–99)	77 (62–164)	0.549

## Data Availability

The data presented in this study are available on request from the corresponding author.
